# Supplementary material for: A new conditional mouse model for Cre-mediated restoration of Hprt1 expression during brain development
Source: bioRxiv. 2026 Sep 17:2026.09.15.751558. Preprint. [Version 1] doi: 10.64898/2026.09.15.751558 (PMC13596364; doi:10.64898/2026.09.15.751558)
Supplement: Supplementary file 1 [file NIHPP2026.09.15.751558v1-supplement-1.pdf]

bioRxiv preprint doi: https://doi.org/10.64898/2026.09.15.751558; this version posted September 17, 2026. The copyright holder for this preprint (which was not certified by peer review) is the author/funder, who has granted bioRxiv a license to display the preprint in perpetuity. It is made available under a CC-BY 4.0 International license.

Supplementary information

Supplementary Table 1. PCR primer sequences.

A. RT-qPCR primer sequences		
Gene	Forward primer ('5 > '3)	Reverse primer ('5 > '3)
ActinB	GAGGTATCCTGACCCTGAAGTA	CACACGCAGCTCATTGTAGA
Ppia	AGCCATGGAGCGTTTTGGGTCC	AGCAGATGGGGTAGGGACGCT
Ywhaz	TAGGTCATCGTGGAGGGTCG	GAAGCATTGGGGATCAAGAACTT
Hprt1 (1)	TTGCTGACCTGCTGGATTAC	GTCCTTTTCACCAGCAAGC
Hprt1 (2)	GTTGGGCTTACCTCACTGCT	TCATCGCTAATCACGACGCT
Hprt1 (3)	CAGTCCCAGCGTCGTGATTA	TGGCCTCCCATCTCCTTCAT

B. PCR primer sequences			
Allele/Gene	Forward primer ('5 > '3)	Reverse primer ('5 > '3)	PCR program
Hprt1^{WT} (298bp) and *Hprt1*$^{\Delta LSL}$ (332bp)	CAGGCCCACCTAGTCAGATAAGAG	CTCACCACGACGCTGGGACT	Hprt1-LSL old
Hprt1^{LSL}	GATCTGCAAGCTAATTCCCGCGG	CTCACCACGACGCTGGGACT	Hprt1-LSL old and Hprt1-LSL new
Hprt1^{WT} (391bp) and *Hprt1*$^{\Delta LSL}$ (425bp)	TGCGGAGTGATTATCTGGGAATCC	CTCACCACGACGCTGGGACT	Hprt1-LSL new
CAGCre	GCTAACCATGTTCATGCCTTC	AGGCAAATTTTGGTGTACGG	CreER
Wnt1-Cre2	CAGCGCCGCAACTATAAGAG	CATCGACCGGTAATGCAG	Wnt1-Cre2
*E2f1**	CTGTTCCAGGGGCTGCATAA	TGACACAGCCCCAAATACCC	Wnt1-Cre2
Sry	TGGGACTGGTGACAATTGTC	GAGTACAGGTGTGCAGCTCT	Sex determination
*IL3**	GGGACTCCAAGCTTCAATCA	TGGAGGAGGAAGAAAAGCAA	Sex determination and CreER

*Positive control gene

bioRxiv preprint doi: https://doi.org/10.64898/2026.09.15.751558; this version posted September 17, 2026. The copyright holder for this preprint (which was not certified by peer review) is the author/funder, who has granted bioRxiv a license to display the preprint in perpetuity. It is made available under a CC-BY 4.0 International license.

Supplementary Table 2. PCR programs.

a. PCR program Hprt1-LSL old

temp	Time (min:sec)	cycles
95,0 °C	3:00	1x
95,0 °C	0:30	35X
59,6 °C	0:30	
72,0 °C	0:45	
72,0 °C	5:00	1X
4,0 °C	∞	1x

b. PCR program Hprt1-LSL new

temp	Time (min:sec)	cycles
95,0 °C	3:00	1x
95,0 °C	0:30	35X
63,0 °C	0:30	
72,0 °C	0:45	
72,0 °C	5:00	1X
4,0 °C	∞	1x

c. PCR program Wnt1-Cre2

temp	Time (min:sec)	cycles
95,0 °C	3:00	1x
95,0 °C	0:30	10X
65-0.5°C*	0:30	
68,0 °C	0:45	
94,0 °C	0:30	30X
60,0 °C	0:30	
72,0 °C	0:45	
72,0 °C	5:00	1X
4,0 °C	∞	1x

* Touchdown protocol starting at 65 with 0.5 °C decrease every cycle down to 60.5 °C.

d. PCR program CreER

temp	Time (min:sec)	cycles
95,0 °C	1:00	1x
95,0 °C	0:30	35X
58,0 °C	1:00	
68,0 °C	0:45	
68,0 °C	5:00	1X
4,0 °C	∞	1x

bioRxiv preprint doi: https://doi.org/10.64898/2026.09.15.751558; this version posted September 17, 2026. The copyright holder for this preprint (which was not certified by peer review) is the author/funder, who has granted bioRxiv a license to display the preprint in perpetuity. It is made available under a CC-BY 4.0 International license.

e. Sex determination

Temp	Time (min:sec)	Cycles
95,0 °C	03:00	1
95,0 °C	00:30	40
58,0 °C	00:30	
72,0 °C	00:45	
72,0 °C	10:00	1
4,0 °C	∞	1

f. RT-qPCR

Temp	Time (min:sec)	Cycles
95,0 °C	02:00	1
95,0 °C	00:05	40
60,0 °C	00:20	
65,0-95,0 °C*	00:05	
4,0 °C	∞	1

* Melt-curve analysis starting at 65 °C with 0.5 °C increase every 5 seconds up to 95 °C.

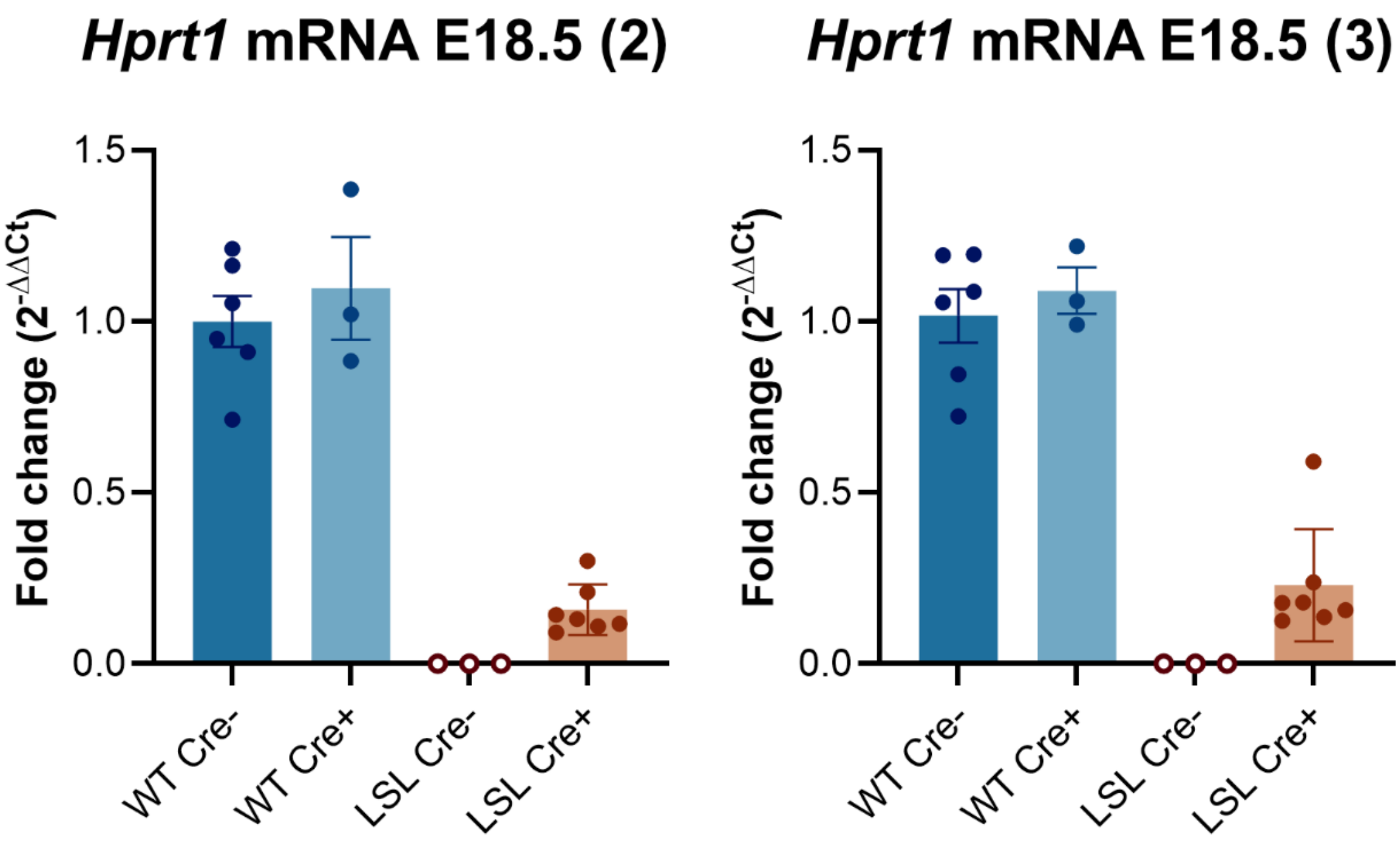

Supplementary Figure 1. Additional qPCR assays support tamoxifen-independent *Hprt1* reactivation. Whole-brain *Hprt1* expression in untreated E18.5 animals measured using two additional primer sets targeting distinct transcript regions. Expression is displayed as $2^{-\Delta\Delta Ct}$ relative to WT Cre−. Group sizes were WT/CreER−, *n* = 6; WT/CreER+, *n* = 3; LSL/CreER−, *n* = 3; and LSL/CreER+, *n* = 7. The same animals were analyzed with both validation assays and the primary assay in Figure 2C; these assays provide technical validation rather than independent biological replication. Bars show group mean ± SEM; points represent individual animals. WT, wild type; LSL, LoxP-mCherry-Stop-LoxP; Cre, CAG-CreER.
